# A time-calibrated phylogeny of the diversification of Holoadeninae frogs

**DOI:** 10.3389/fbinf.2024.1441373

**Published:** 2024-10-02

**Authors:** Júlio C. M. Chaves, Fábio Hepp, Carlos G. Schrago, Beatriz Mello

**Affiliations:** ^1^ Departamento de Genética, Universidade Federal do Rio de Janeiro, Rio de Janeiro, Brazil; ^2^ Laboratório de Anfíbios e Répteis, Departamento de Zoologia, Universidade Federal do Rio de Janeiro, Rio de Janeiro, Brazil; ^3^ Departamento de Vertebrados, Museu Nacional, Universidade Federal do Rio de Janeiro, Rio de Janeiro, Brazil

**Keywords:** Anura, Neobatrachia, timetree, divergence times, MCMCTree

## Abstract

The phylogeny of the major lineages of Amphibia has received significant attention in recent years, although evolutionary relationships within families remain largely neglected. One such overlooked group is the subfamily Holoadeninae, comprising 73 species across nine genera and characterized by a disjunct geographical distribution. The lack of a fossil record for this subfamily hampers the formulation of a comprehensive evolutionary hypothesis for their diversification. Aiming to fill this gap, we inferred the phylogenetic relationships and divergence times for Holoadeninae using molecular data and calibration information derived from the fossil record of Neobatrachia. Our inferred phylogeny confirmed most genus-level associations, and molecular dating analysis placed the origin of Holoadeninae in the Eocene, with subsequent splits also occurring during this period. The climatic and geological events that occurred during the Oligocene-Miocene transition were crucial to the dynamic biogeographical history of the subfamily. However, the wide highest posterior density intervals in our divergence time estimates are primarily attributed to the absence of Holoadeninae fossil information and, secondarily, to the limited number of sampled nucleotide sites.

## Introduction

In recent decades, numerous attempts have been made to elucidate the phylogenetic relationships within the Amphibia (Frost et al., 2006; Hedges et al., 2008; Pyron and Wiens, 2011; Hime et al., 2021; Portik et al., 2023). The evolutionary relationships among major amphibian lineages remain contentious. Most studies have tackled this problem by maximizing taxonomic coverage and inferring large-scale phylogenies from limited gene sets, which have yielded distinct relationships compared to analyses that expanded gene sampling (Wiens et al., 2010; Pyron and Wiens, 2011; Feng et al., 2017; Jetz and Pyron, 2018; Streicher et al., 2018; Hime et al., 2021; Portik et al., 2023). Additionally, studies have also estimated timescales for large anuran groups (Roelants et al., 2007; Pyron, 2014; Feng et al., 2017; Jetz and Pyron, 2018; Hime et al., 2021; Portik et al., 2023; Frazão et al., 2015). However, little focus has been given to elucidating the evolutionary scenario that occurred within the families, as studies have mainly concentrated on a few genera or used a limited number of species and loci.

The South American subfamily Holoadeninae Hedges et al., 2008, which comprises 73 frog species distributed across nine genera within Strabomantidae (Miranda-Ribeiro, 1920; Barbour, 1930; Griffiths, 1959; Heyer, 1969; Frost et al., 2006; Hedges et al., 2008; De La Riva et al., 2018; Catenazzi et al., 2020; Dubois et al., 2021), is an anuran lineage with a scarce number of molecular phylogenetic studies. Holoadeninae species are small (14–48 mm of snout-vent length) direct-developing frogs (Hedges et al., 2008; Vitt and Caldwell, 2014). Notably, the subfamily exhibits a disjunct geographical distribution across diverse South American biomes, encompassing the Brazilian Atlantic coastal rainforest, the Brazilian savanna (known as the ‘Cerrado’), the Andes of Peru, Ecuador and Bolivia, and lowlands in Amazonia, Ecuador, and Colombia (Frost et al., 2006). Recent works focusing on species description inferred molecular phylogenies for subclades within this subfamily (De La Riva et al., 2018; Venegas et al., 2018; Reyes-Puig et al., 2019; Santa-Cruz et al., 2019; Catenazzi et al., 2020; Motta et al., 2021). However, no consensus has been reached on the Holoadeninae phylogeny, although some evolutionary relationships are frequently recovered, such as the grouping of *Euparkerella* + *Holoaden*, and *Bahius* + (*Barycholos* + *Noblella*) (Venegas et al., 2018; Motta et al., 2021).

Because Holoadeninae lacks a fossil record, no timescale specifically focused on the group was ever estimated (Sanchiz and Rocek, 1996; Agnolin et al., 2020; Barcelos and Dos Santos, 2023). For instance, the TimeTree database (Kumar et al. (2022), last accessed on 16 May 2024) lists only 14 Holoadeninae species and 11 divergences within the group, most of them dated in a single study (Pyron, 2014). This, coupled with the absence of a taxonomic representative phylogeny, makes it difficult to construct an evolutionary scenario for this lineage, which is crucial for studying the evolution of particular phenotypes, such as miniaturization, which appears to have evolved independently in this group (Duellman and Lehr, 2009; Fusinatto et al., 2013; Santa-Cruz et al., 2019). A potential solution is to use alternative strategies for molecular dating analysis, such as broadening taxonomic sampling to include target calibration nodes. In this study we implemented this approach by using a comprehensive molecular dataset to infer the phylogenetic relationships and divergence times of the Holoadeninae subfamily. We have increased the number of divergence dates available at TimeTree database (http://www.timetree.org), expanding the timetree of this lineage, providing a more complete scenario for the diversification of this group.

## Methods

### Dataset assembly

We accessed the list of Holoadeninae species through the *Amphibian Species of the World* reference website (https://amphibiansoftheworld.amnh.org), which yielded a total of 73 species. To maximize loci coverage, we selected representative lineages from the same family (Strabomantidae) as outgroups. Additionally, we included non-Strabomantidae species for inclusion of fossil calibration information. After a thorough search in the NCBI database (Sayers et al., 2019), we selected six genes widely available for Holoadeninae species: three nuclear loci (RAG1, TYR, and POMC) and three mitochondrial loci (12S, 16S, and COI). The final matrix consisted of 63 species, comprising 49 Holoadeninae and 15 outgroups. The complete species list and GenBank accession numbers are provided in the (Supplementary Table S1). In this dataset, hereafter referred to as “DM”, the average number of loci per species was 3.75 (62.5%), indicating a significant proportion of missing data. To address this, we also compiled two smaller datasets with higher levels of completeness. Dataset “D3” contained only those terminals with at least three loci (50%), resulting in 49 species. Dataset “D4” comprised 40 species that presented at least four loci (66.7%).

### Phylogenetic inference

Each gene was aligned individually across all datasets. Coding genes (RAG1, TYR, POMC, and COI) were aligned in the SeaView software (Gouy et al., 2010), based on the amino acid sequences, using Clustal with default options (Thompson et al., 1994). Ribosomal genes 12S and 16S were aligned in the online platform T-coffee (Notredame et al., 2000), using the secondary structure (R-Coffee) option. Gene trees were inferred using IQ-TREE2 software (Nguyen et al., 2015), with 1,000 UFBoot replicates (Minh et al., 2013; Hoang et al., 2018) for each gene across all datasets. The best-fit substitution models were inferred using ModelFinder (Kalyaanamoorthy et al., 2017). To account for incomplete lineage sorting, we used ASTRAL (Mirarab and Warnow, 2015) to estimate species phylogeny from the gene trees generated in IQ-TREE2. Poor supported branches (UFBoot <30%) were collapsed for each gene tree to avoid the recovery of high-supported unreliable clades. Statistical support for branches of the ASTRAL tree was accessed by the quartet support (Q1). For the sake of comparison, concatenated analyses for all datasets were also carried out in IQ-TREE2, following the same procedure.

### Molecular dating

We selected dataset D3 for the molecular dating analysis because the DM dataset, which had a high frequency of missing data, resulted in a polyphyletic Holoadeninae. While dataset D4 recovered a monophyletic Holoadeninae, it included fewer species and thus did not represent most Holoadeninae diversity. Dated phylogenies were inferred using the RelTime method in MEGA X (Kumar et al., 2018) and in MCMCTree (Yang and Rannala, 2006), employing the ASTRAL tree, the concatenated alignment and the GTR + G substitution model (Tavaré, 1986; Yang, 1994). Calibration points were chosen based on the fossil record of Neobatrachia.

I. The root of the phylogenetic tree, representing the split between the outgroup (*Rana temporaria*) and Nobleobatrachia in our dataset, was assigned a minimum age of 100.5 Ma based on the fossil *Cratia gracilis* (Báez et al., 2009), and a maximum age of 161.2 Ma based on the fossil *Rhadinosteus parvus* (Henrici, 1998).
II. The split of Leptodactylidae and the remaining Nobleobatrachia species was calibrated with a maximum age of 125 Ma, based on the fossil *Eurycaphalella alciane* (Báez et al., 2009).
III. The crown node of Terrarana was calibrated with a minimum age of 33.9 Ma, based on the fossil *Eleutherodactylus* sp. (Poinar and Cannatella, 1987).
IV. The split between and *Chacophrys* and *Ceratophrys* was calibrated with a minimum constraint of 9.07 Ma, based on the age of the fossil *Ceratophrys* sp. (Barcelos and Dos Santos, 2023).

This calibration scheme is referred as calibration scenario 1. However, given the significant impact that maximum calibration constraints can have on final time estimates, we conducted molecular dating analyses exploring additional calibration scenarios, referred to here as calibration scenarios 2 and 3. In scenario 2, calibration point I was adjusted to have a maximum age of 185.5 Ma, based on the upper limits of the confidence intervals for the divergence between Neobatrachia and Pelobatoidea as provided in recent studies (Portik et al., 2023; Hime et al., 2021). Additionally, for calibration point II, a maximum constraint of 83 Ma was adopted, also based on the upper limit of the confidence interval for the Hyloidea crown node from the same studies. In calibration scenario 3, calibration point I remained the same as in scenario 2, but calibration point II was removed from the analysis due to some recent studies suggesting an older origin for crown Hyloidea (*e.g.*, Jetz and Pyron, 2018). Calibration points III and IV were consistent across all scenarios.

Because RelTime does not assume prior distributions to model rate evolution and divergence times, and is a fast molecular dating method, it is a valuable tool for investigating the impact of calibrations on divergence time estimation (Battistuzzi et al., 2015). Therefore, we ran RelTime without calibrations to infer relative divergence times. Absolute divergence times were inferred in MCMCTree. In this case, the minimum and maximum times adopted as time constraints for calibration were used as boundaries to delimit uniform prior distributions with soft bounds (left and right tail probabilities equal 0.025). Calibration point II was incorporated as a maximum bound prior (right tail probability of 0.025), except in calibration scenario 3, where this calibration was not used. Calibration points III and IV were informed as minimum bound priors (left tail probabilities equal 0.025). The time unit was set to 100 million years. To derive a rate estimate to use as the prior mean for the overall rate parameter (rgene_gamma = 0.274413 1), we ran baseml under the global clock model with calibration information. The rate drift parameter was “sigma2_gamma = 1 1” and the parameters of the birth-death process were “BDparas = 2.828156 1.487060 1.000000”, retrieved using the approach described in Tao et al. (2021). Markov chain Monte Carlo (MCMC) was sampled every 100th generation until effective sample size (ESS) values were higher than 200 (after removing the burn-in period accordingly). Divergence times were estimated using both autocorrelated and uncorrelated rate evolution models, using the multivariate normal approximation (Reis and Yang, 2011). We carried out the analysis twice under each rate evolution model to ensure the convergence of the chains.

To estimate the extent to which the uncertainty associated with divergence time estimates was impacted by the number of sampled nucleotide sites or the variance of calibration priors, we also conducted an infinite sites analysis using PAML’s infinitesites software. This analysis estimates divergence times under the theoretical expectation of infinitely long sequences. This approach is useful for determining whether additional data would reduce the uncertainty of divergence time estimates or if only the inclusion of additional fossil calibrations can make estimates more precise. The infinite sites theory predicts a linear relationship between divergence time estimates and the widths of the highest posterior densities (HPDs) of these estimates (Yang and Rannala, 2006; Rannala and Yang, 2007). Using results from both the original and infinite sites analyses, we built linear models in which the width of the HPD intervals was the response variable (*y*) and their respective estimated node ages were entered as features (*x*). The β coefficient of the regression line crossing the origin, y=βx , measures how the uncertainty of age estimates are related to divergence times.

## Results

**FIGURE 1:**
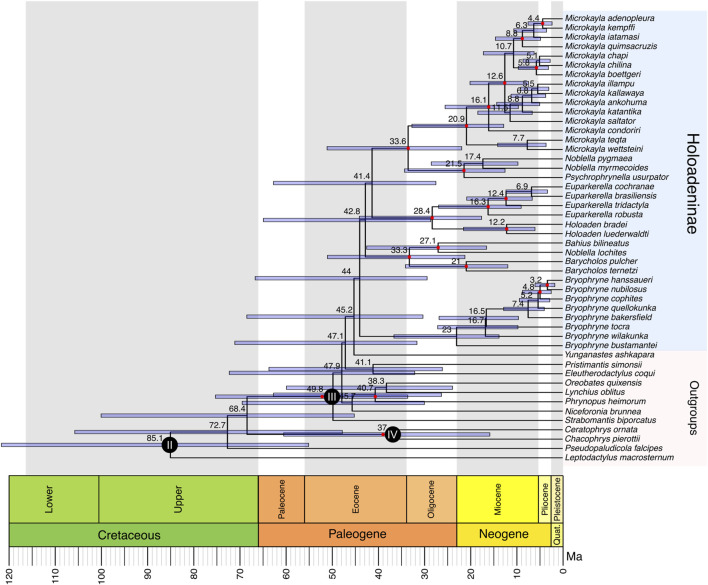
Holoadeninae Timetree estimated in MCMCTree using a correlated rates model and four fossil calibration points (calibration scenario 1). Calibrated nodes are indicated with a dark-filled circle (calibration I is not shown because it corresponds to the divergence of the outgroup species, *Rana temporaria*, which was removed from the figure). The numbers inside the circles correspond to the calibration numbers defined in the methods section. Filled red circles indicate nodes that had Q1 support values from ASTRAL ≥0.75. Divergence time estimates are shown in black to the left of each node.

The tree inferred by ASTRAL using the D3 dataset was selected as the most reliable and is depicted in Figure 1. This choice was made because 1) the monophyly of Holoadeninae was recovered, and 2) the number of species was higher than in the D4 dataset, which also led to a monophyletic Holoadeninae. Therefore, the results and discussion will consider the phylogenetic hypothesis and divergence time estimation recovered from the D3 dataset analyzed in ASTRAL. The inferred phylogenies based on the DM and D4 datasets, using IQ-TREE and ASTRAL, are included in the Supplementary Material.

Holoadeninae monophyly was recovered with low support value (Q1 = 0.6). Within Holoadeninae, only the genus *Noblella* was not recovered as monophyletic: *N. myermecoides* and *N. pygmaea* were recovered as a sister group to *Psychrophrynella*, while *N. lochites* was recovered as sister to *Bahius*. The first lineage to diverge within the subfamily was composed of *Bryophryne* species, forming a monophyletic clade with low support (Q1 = 0.48). The second lineage to diverge was composed of *Barycholos* species, *Noblella lochites*, and *Bahius bilineatus*, which presented high support value (Q1 = 0.89). *Barycholos* species formed a monophyletic clade with high support value (Q1 = 1.0). *Noblella lochites* and *Bahius bilineatus* grouped together with low support value (Q1 = 0.74). The lineage composed of *Barycholos* species, *N*. *lochites* and *Bahius bilineatus* was the sister group of a clade including *Holoaden*, *Euparkerella, Psychrophrynella*, *Noblella myrmecoides*, *Noblella pygmea*, and *Microkayla*. All these species grouped together with low support (Q1 = 0.37). *Euparkerella* and *Holoaden* species formed a monophyletic clade with high support (Q1 = 0.97), which was the sister lineage of the group comprised of *Psychrophrynella*, *Noblella myrmecoides*, *Noblella pygmea*, and *Microkayla*. The support for the clade containing *Holoaden*, *Euparkerella, Psychrophrynella*, *Noblella myrmecoides*, *Noblella pygmea*, and *Microkayla* species was low (Q1 = 0.63). The genus *Euparkerella* was monophyletic (Q1 = 1.0), as well as *Holoaden* (Q1 = 0.8). *Microkayla* species comprised a monophyletic lineage (Q1 = 0.99) that was the sister group of *Psychrophrynella usurpator* + (*N. myrmecoides* + *N. pygmea*) with high support (Q1 = 0.84). *Noblella myrmecoides* and *N. pygmea* grouped together with low support (Q1 = 0.67) and formed the sister lineage of *P. usurpator* (Q1 = 0.88). The final normalized quartet score from ASTRAL was 0.82, indicating a high level of discordance among the gene trees.

Molecular dating analyses obtained in MCMCTree under distinct calibration schemes and rate evolution models indicated that the autocorrelated rates model produced more congruent divergence time estimates across the distinct calibration scenarios (Supplementary Figure S7). Calibration scenarios 1 and 3 showed congruence under an autocorrelated rates model (Slope = 1.03), while scenarios 1 and 2 displayed a lower correspondence (Slope = 0.84). When using an uncorrelated rates model, the correspondence between the different calibration scenarios decreased. In this case, the estimated divergence times under calibration scenario 3 were generally about 12% younger than those under calibration scenario 1 (Slope = 0.88). Calibration scenario 2 produced even younger time estimates, approximately 24% younger than those in scenario 1 (Slope = 0.760). These results highlight that the timetree obtained using a maximum constraint of 83 Ma to calibrate the divergence between Leptodactylidae and other Nobleobatrachia (calibration scenario 2) should be viewed with caution, as it led to much younger node ages. While it is possible that divergence times are indeed younger, we believe that a more conservative approach is more appropriate. Additionally, relaxing the maximum constraint used to calibrate the root of the phylogenetic tree (calibration scenario 3) had little impact on time estimates obtained under a correlated rates model in MCMCTree. This, combined with the finding that rate correlation produced node ages more congruent with relative uncalibrated divergence times (see below), suggested that the divergence times estimated using correlated rates and calibration scenario 1 in MCMCTree was the most robust.

**FIGURE 2:**
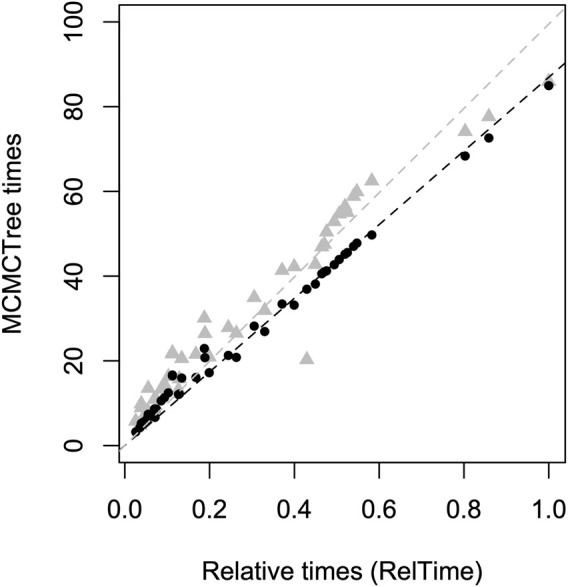
Comparison of RelTime relative time estimates (without using calibrations, *x*-axis) and MCMCTree absolute time estimates (using Calibration Scenario 1, *y*-axis). MCMCTree estimates using a correlated rate evolution model are represented by black dots, while estimates using an uncorrelated rates model are depicted by gray triangles. The dashed lines indicate the linear regressions through the origin, considering correlated rates (black, R^2^ = 0.99) and uncorrelated rates (gray, R^2^ = 0.97).

The relative divergence times obtained with RelTime (without calibration constraints) and the absolute node ages from MCMCTree under an autocorrelated clock were highly correlated (*R* ^2^ = 0.99, *p* < 0.01) (Figure 2). In contrast, the relationship between RelTime relative times and MCMCTree estimates under an uncorrelated clock showed a lower correlation (*R* ^2^ = 0.97, *p* < 0.01). Therefore, due to the independent convergence between RelTime and MCMCTree autocorrelated results, combined with the results that indicated that autocorrelated rates produced more congruent divergence time estimates across the distinct calibration scenarios, all time estimates referred from now on will relate to the MCMCTree autocorrelated analysis (RelTime and MCMCTree results under an uncorrelated rates model are included in the Supplementary Material). Infinite sites analysis indicated that sequencing additional nucleotide sites would be unlikely to increase the precision of divergence time estimates. The width of HPDs decreased by 2.0% of the estimated node ages when comparing the inference based on the D3 alignment and the inference under the infinite sites assumption (both using calibration scenario 1). This indicates that sampling error accounted for only 2.0% of the uncertainty in divergence times. Thus, reducing HPD intervals will require additional and accurate calibrations.

Holoadeninae diversification was dated at 44.0 Ma (29.3–66.6 Ma). The time of the most recent common ancestor (tMRCA) of *Bryophryne* species was dated around 23.0 Ma (13.7–36.5 Ma). *Barycholos* + (*Noblella lochites* + *Bahius bilineatus*) divergence from the remaining Holoadeninae lineages was inferred as 42.8 Ma (28.5–64.8 Ma). The tMRCA of the group composed of *Barycholos* + (*Noblella lochites* + *Bahius bilineatus*) was estimated at 33.3 Ma (21.1–50.9 Ma). The split between *Bahius* and *Noblella lochites* occurred at 27.1 Ma (16.4–42.4 Ma). The divergence between the two *Barycholos* species was dated at 21.0 Ma (11.8–34.0 Ma). The tMRCA of the clade containing *Holoaden*, *Euparkerella*, *Psychrophrynella*, *Noblella myrmecoides*, *Noblella pygmea*, and *Microkayla* was estimated at 41.4 Ma (27.4–62.6 Ma). *Holoaden* and *Euparkerella* diverged at 28.4 Ma (17.5–44.0 Ma). *Holoaden bradei* and *H. luederwaldti* shared a common ancestor at 12.2 Ma (5.9–21.4 Ma), while *Euparkerella* species diversified at 16.3 Ma (8.9–26.8 Ma). The tMRCA of *Psychrophrynella*, *Noblella myrmecoides*, *Noblella pygmea*, and *Microkayla* was dated at 33.6 Ma (21.8–51.0 Ma). The split between *Psychrophrynella* and *Noblella myrmecoides + Noblella pygmea* was inferred at 21.5 Ma (12.4–34.2 Ma). *Noblella myrmecoides* and *N. pygmaea* species separated 17.4 Ma (9.6–28.4 Ma). *Microkayla* diversification was estimated at 20.9 Ma (12.7–32.6 Ma).

## Discussion

This study has inferred the most taxon- and loci-comprehensive timetree of Holoadeninae, a Neotropical frog subfamily, including representative species from all its genera, except for *Qosqophryne*. Divergence time estimation was based on fossil calibrations, providing an expanded timetree for this group, as the number of dated divergence events has considerably increased. The tMRCA of Holoadeninae was placed within the Eocene period, contrasting with most previous studies that focused on inferring the timescale of higher amphibian taxa (*e.g.*, Gomez-Mestre et al., 2012; Pyron, 2014; Hedges et al., 2015). Despite the wide HPD intervals, we were able to allocate the origin and diversification of most genera along the Miocene, a period marked by significant geological and climatic changes in South America (Hoorn et al., 2010; Latrubesse et al., 2010).

Our inferred tree topology generally aligns with the genus-level relationships recently recovered by Jetz and Pyron (Jetz and Pyron, 2018). Except for COI, all genes employed in the present study were included in Jetz and Pyron (2018)’s analysis, which may account for the similarity in results. However, although both studies inferred a close relationship between *Bahius*, *Barycholos* and some *Noblella* species (since this genus may be paraphyletic), we found *Bryophryne* as the first lineage to diverge within this group, whereas Jetz and Pyron (2018) retrieved the group comprising *Bahius*, *Barycholos* and some *Noblella* species as the first splitting lineage. Other studies have found genus-level phylogenetic relationships distinct from our results (*e.g.*, Hedges et al., 2008; Pyron and Wiens, 2011; Canedo and Haddad, 2012; Padial et al., 2014; De La Riva et al., 2018; Heinicke et al., 2018; Venegas et al., 2018; Reyes-Puig et al., 2019; Santa-Cruz et al., 2019; Catenazzi et al., 2020; Motta et al., 2021). Several factors could explain these discrepancies, such as lower species coverage (Hedges et al., 2008; Pyron and Wiens, 2011; Canedo and Haddad, 2012; Padial et al., 2014) or the use of different loci (Pyron and Wiens, 2011; Padial et al., 2014; De La Riva et al., 2018; Jetz and Pyron, 2018; Venegas et al., 2018; Motta et al., 2021; Portik et al., 2023).

The most recent molecular phylogeny estimated for Holoadeninae is the hypothesis proposed by Portik et al. (2023), as part of a large timetree inferred for frogs (Portik et al., 2023). The genus-level phylogenetic relationships recovered by this study differs from ours. Our results showed *Bryophryne* as the first lineage to diverge within Holoadeninae, while Portik et al. (2023) recovered this genus as related to *Microkayla*, *Psychrophrynella* and some *Noblella* species. Another difference was that we estimated *Euparkerella + Holoaden* as the sister lineage of a clade composed by *Psychrophrynella*, *Noblella myrmecoides*, *Noblella pygmea*, and *Microkayla*. In contrast, Portik et al. (2023) inferred *Euparkerella + Holoaden* as the sister clade of *Barycholos* and some *Noblella* species. However, both studies found low support values for these relationships. While Portik et al. (2023) included a few more Holoadeninae species than our study (51 compared to the 36 species we sampled), their alignment matrix was highly incomplete regarding Holoadeninae, with most species presenting more than 90% of missing data.

Importantly, low ASTRAL support values indicated that most phylogenetic relationships within the subfamily are yet to be resolved (Figure 1). The normalized quartet support from the ASTRAL analysis (0.82) was higher than the recommended cutoff value (0.75) (Mirarab, 2019; Rabiee et al., 2019), though the difference was not substantial. These results suggest that incomplete lineage sorting (ILS) may have been pervasive during the early diversification of Holoadeninae. In fact, the estimated timetrees, regardless of the rate evolution model and calibration scenario used, indicated that the early divergences within the subfamily occurred within a very narrow timeframe, of less than 10 million years. Such short intervals between speciation events are known to increase levels of ILS (Maddison, 1997; Maddison and Knowles, 2006; Degnan and Rosenberg, 2009; Edwards, 2009), which might explain the poorly supported relationships.

Low supports for Holoadeninae divergences were retrieved in previous molecular phylogenetic studies (*e.g.*, Canedo and Haddad, 2012; Reyes-Puig et al., 2019; Portik et al., 2023). However, two phylogenetic relationships are repeatedly recovered and highly supported across several works, namely, (i) the grouping of *Euparkerella* and *Holoaden* (Canedo and Haddad, 2012; De La Riva et al., 2018; Jetz and Pyron, 2018; Reyes-Puig et al., 2019; Santa-Cruz et al., 2019; Motta et al., 2021; Portik et al., 2023); and (ii) the paraphyly of *Noblella* (Hedges et al., 2008; Pyron and Wiens, 2011; Canedo and Haddad, 2012; Padial et al., 2014; De La Riva et al., 2018; Jetz and Pyron, 2018; Portik et al., 2023). Our results suggested a close evolutionary affinity between some *Noblella* species and *Psychrophrynella*, as previous works did (*e.g.*, Reyes-Puig et al., 2020; Portik et al., 2023). Additionally, we estimated the relatedness of *Microkayla*, *Psychrophrynella*, and some *Noblella* species, a finding that has been previously reported in other studies (Santa-Cruz et al., 2019; Catenazzi et al., 2020; Reyes-Puig et al., 2020; Motta et al., 2021; Portik et al., 2023).

The mean of the posterior distribution of node ages in our inferred timescale suggests that Holoadeninae originated during the Eocene, followed by a period of rapid diversification. Large-scale studies that estimated divergence times for higher taxa within Amphibia have generally obtained ancient ages for the origin of this subfamily, placing it in the Paleocene (Gomez-Mestre et al., 2012; Pyron, 2014; Hedges et al., 2015). In contrast, studies focusing on lower-taxa have retrieved a younger origin, in the Eocene (Heinicke et al., 2007; Gonzalez-Voyer et al., 2011; Fouquet et al., 2022). The timetree presented in Figure 1 supports a more recent origin, with MCMCTree HPD indicating that the tMRCA of Holoadeninae existed between 29.3 and 66.6 Ma. This result is in agreement with the recent study of Portik et al. (2023), which focused on the timetree of anuran higher taxa (Portik et al., 2023). However, our estimated times for the diversification of Holoadeninae genera were generally older than those provided by Portick et al. (2023).

The absence of a fossil record may be driving the different tMRCAs estimated for Holoadeninae. Most studies conducted so far have used limited loci sampling or largely incomplete alignment matrices, which can make the estimated divergence times sensitive to taxon sampling and the markers chosen. Due to the lack of temporal information to adjust rates and infer times, the final estimated times are more susceptible to methodological artifacts. It is also important to note that the divergence time estimates we obtained in MCMCTree using an uncorrelated rate prior led to older node ages, making them closer to the Paleocene origin suggested by some studies (Figure 2). In this case, Holoadeninae tMRCA was dated at 54.7 Ma (HPD 34.9–78.8 Ma). However, two factors argue against this scenario: 1) the weaker correlation between RelTime relative time estimates and those obtained by MCMCTree using an uncorrelated rates prior, and 2) the higher sensitivity of time estimates to different calibration scenarios when using the uncorrelated rates model.

The inferred Holoadeninae timetree indicates that genera may have diversified mainly throughout the Miocene. A Miocene diversification is a pattern also observed in other South American frog lineages (Santos et al., 2009; Castroviejo-Fisher et al., 2014; Réjaud et al., 2020; Ortiz et al., 2023). Regarding the colonization of the Atlantic Forest, our results support two possible scenarios, both occurring during the Oligocene-Miocene transition. During this period, significant geological changes took place in South America, including shifts in temperature and humidity that favored the expansion of the Neotropical rainforests (Jaramillo et al., 2006; Hoorn et al., 2010; Jaramillo, 2023). The first scenario involves two independent colonizations of the Atlantic Forest: one by the ancestor of *Holoaden* and *Euparkerella*, and another involving *Bahius bilineatus* (though the placement of this species is not well supported). Evidence suggests that several vertebrate lineages colonized the Atlantic Forest via biotic interchanges routes stablished as early as the Upper Oligocene between the Amazon and the Brazilian Atlantic coastal rainforest (Ledo and Colli, 2017; Prates et al., 2017; Pirani et al., 2020), supporting this scenario. The second possibility is that ancestral lineages of Holoadeninae were distributed continuously from east to west South America, and after the formation of the Brazilian savannah (Cerrado) in the Oligocene-Miocene transition, the ancestors of *Holoaden* + *Euparkerella* and of *Bahius billineatus* remained in the Brazilian Atlantic coastal rainforest, as well as the ancestor of *Barycholos ternetzi* in the Cerrado, as relictual lineages. In this case, vicariance events would explain the disjunct distribution of Holoadeninae. The currently broad South American distribution of *Barycholos*, from the Pacific coast and lowlands of Ecuador to the central regions of Brazil, reinforces this latter hypothesis. Future biogeographical studies are needed to shed more light on these historical events.

Our study presents a timetree for the Holoadeninae subfamily, shedding light on the evolutionary history of this diverse group of Neotropical frogs. Our divergence time estimates suggest that Holoadeninae originated during the Eocene and that genera diversified primarily throughout the Miocene, an epoch marked by significant geological and climatic changes in South America. Additionally, the climatic and geological events that occurred during the Oligocene-Miocene transition likely played a significant role in the disjunct distribution of the subfamily. Although some phylogenetic relationships remain unresolved due to low support values, certain genus-level relationships were consistently recovered across various studies, highlighting their robustness. We demonstrated that the wide HPD intervals were primarily due to poor fossil information. Therefore, new fossil discoveries would enhance the precision of the Holoadeninae timescale. Nevertheless, future efforts to generate genomic data for species within this subfamily are highly valuable, as genome-wide data enables sophisticated phylogenetic analyses under the multispecies coalescent framework. This approach will not only improve our understanding of the biological aspects of this neglected frog lineage but also provide deeper insights into the evolutionary processes that have shaped Holoadeninae diversity and geographical distribution.

## Data Availability

The original contributions presented in the study are included in the article/Supplementary material, further inquiries can be directed to the corresponding author.
