# Supplementary material for: A time-calibrated phylogeny of the diversification of Holoadeninae frogs
Source: Front Bioinform. 2024 Oct 2;4:1441373. doi: 10.3389/fbinf.2024.1441373 (PMC11480671; doi:10.3389/fbinf.2024.1441373)
Supplement: Supplementary file 1 [file DataSheet1.PDF]

Supplementary Material

A time-calibrated phylogeny of the diversification of Holoadeninae frogs

Júlio C. M. Chaves[1,2], Fábio Hepp[2,3], C. G. Schrago[1] and Beatriz Mello[1]*

[1] Departamento de Genética, Universidade Federal do Rio de Janeiro, Brazil
[2] Laboratório de Anfíbios e Répteis, Departamento de Zoologia, Universidade Federal do Rio de Janeiro, Brazil
[3] Departamento de Vertebrados, Museu Nacional, Universidade Federal do Rio de Janeiro, Brazil

- GenBank accession numbers

Table S1: NCBI accession numbers for the sequences used in this study.

Species		*Tyr*	*COI*	*POMC*	*RAG1*	*12S*	*16S*
Barycholos	*ternetzi*	JX267680	KU494358.1	JX298136	JX298183	MT992087.1	JX267466.1
Euparkerella	*brasiliensis*	JX298237.1	KU494434	JX298137	JX298185	JX298276	JX267468
Holoaden	*bradei*	EU186779	JX298358.1	JX298138	JX298186	EF493378.1	EF493366.1
Microkayla	*chilina*	MF186561.1	MF186457	MF186496	MF186539.1	MF186327	MF186414
Microkayla	*iatamasi*	MF186558.1	MF186461	MF186511	MF186536	MF186301.1	MF186368
Microkayla	*katantika*	MF186576	MF186453.1	MF186491.1	MF186533	MF186307	MF186381.1
Bahius (Heyerus)	*bilineatus*	MW203020.1			MW201169.1	MW201163	MW201163
Barycholos	*pulcher*	EU186765			KX208662	EU186727	EU186709
Bryophryne	*tocra*	MF186583		MF186522	MF186541	MF186315	MF186398.1
Bryophryne	*bakersfield*		MF186452	MF186525	MF186528.1	MF186287.1	KT276287.1
Bryophryne	*bustamantei*	MF186581.1		MF186524	MF186543.1	MF186296	MF186356
Bryophryne	*cophites*	KY681062.1	KY672976		KY672961	EF493537.1	KY652641
Holoaden	*luederwaldti*	EU186768.1	KU494455.1		JX267549	EU186728	EU186710
Microkayla	*adenopleura*	MF186565.1		MF186487	MF186537.1	MF186283	MF186340
Microkayla	*kempffi*	MF186566.1		MF186504	MF186538	MF186308.1	MF186384
Microkayla	*wettsteini*	MF186551		GQ345266	MF186531	MF186338	MF186434.1
Microkayla	*chapi*	MF186562		MF186481.1	MF186540.1	MF186328	MF186417
Microkayla	*condoriri*	MF186550.1		MF186480	MF186530	MF186300	MF186360
Microkayla	*boettgeri*	MF186559	MF186456	MF186484		MF186294	MF186353
Microkayla	*illampu*	MF186549.1		MF186510.1	MF186534.1	MF186305	MF186369.1
Noblella	*pygmaea*	KY681066	KY672979		KY681086		KY652645
Bryophryne	*hanssaueri*	KY681063	KY672977		KY681084		KY652642
Bryophryne	*nubilosus*	KY681064	KY672978		KY681085		KY652643
Bryophryne	*quellokunka*			MF186479	MF186526.1	MF186309	MF186387.1

Microkayla	*ankohuma*	MF186560		MF186509		MF186288	MF186346
Microkayla	*kallawaya*	MF186575		MF186507.1		MF186306	MF186376
Microkayla	*teqta*	MF186552.1		MF186472		MF186322	MF186404
Noblella	*myrmecoides*	KY681065.1			KY672962	JX267464	JX267542
Noblella	*lochites*	EU186777			EU186756	EU186699.1	EU186699.1
Psychrophrynella	*usurpator*	KY681083	KY672994.1		KY672975		KY652662
Bryophryne	*wilakunka*			MF186520		MF186291	MF186349.1
Euparkerella	*cochranae*	KF625112.1	KF625055.1		KF625090.1		
Euparkerella	*robusta*	KF625111	KF625054		KF625089		
Euparkerella	*tridactyla*	KF625109.1	KF625053		KF625087.1		
Microkayla	*quimsacruzis*			MF186505		MF186323	MF186407
Microkayla	*saltator*			MF186508.1		MF186326	MF186410
Microkayla	*chacaltaya*				MF186532		MF186357.1
Microkayla	*illimani*			MF186482			MF186374
Noblella	*heyeri*					JX267463	JX267541.
Noblella	*peruviana*					EF493714.1	EF493714.1
Bryophryne	*phuyuhampatu*						MF419254
Noblella	*losamigos*		MN355545				
Noblella	*madreselva*				MN355547		
Noblella	*personina*				MN068028.1		
Psychrophrynella	*chirihampatu*						KU884559
Psychrophrynella	*glauca*						MG837565
Qosqophryne	*flammiventris*				MT431913		
Qosqophryne	*gymnotis*				MT431914		
Qosqophryne	*mancoica*		MT435519.1				
Phrynopus	*heimorum*	MF186580	MF186462	MF186515	MF186545	MF186363.1	MF186302.1
Oreobates	*quixensis*	JF809893	JX298360.1		MZ560987.	EF493662	EF493828
Lynchius	*oblitus*	KX470802			KX470793	KX470782	KX470775
Yunganastes	*ashkapara*	JF809898			JF809919	EU192233	FJ438807
Niceforonia	*brunnea*	EF493484		GQ345264	KX208676		
Pristimantis	*simonsii*			AY819155		AM039641	AM039709
Strabomantis	*biporcatus*	EU186775		GQ345265	EU186754		
Serranobatrachus	*sanctaemartae*		OP787204.1				
Tachiramantis	*letinginosus*	KP297390					
Eleutherodactylus	*coqui*		KY033486.1		EF107341.1	GQ345176.1	GQ345176.1
Ceratophrys	*ornata*	KP295675.1	KP295693.1	KP295571.1		JX564858.1	JX564858.1
Chacophrys	*pierottii*	KP295677.1	KP295695.1			KP295621.1	KP295621.1
Leptodactylus	*macrosternum*	MT496576.1	KU494550.1	MT496683.1	MT996125.1	KM091484.1	KM091598.1
Pseudopaludicola	*falcipes*	KT882525.1	KC520684.1	KC604051.1	HQ634171.1	KJ146972.1	KJ146972.1
Rana	*temporaria*	KC800238.1	MN993213.1	KC800313.1	KX269561.1	MT483700.1	MT483700.1

- Phylogenies inferred with dataset DM and D4

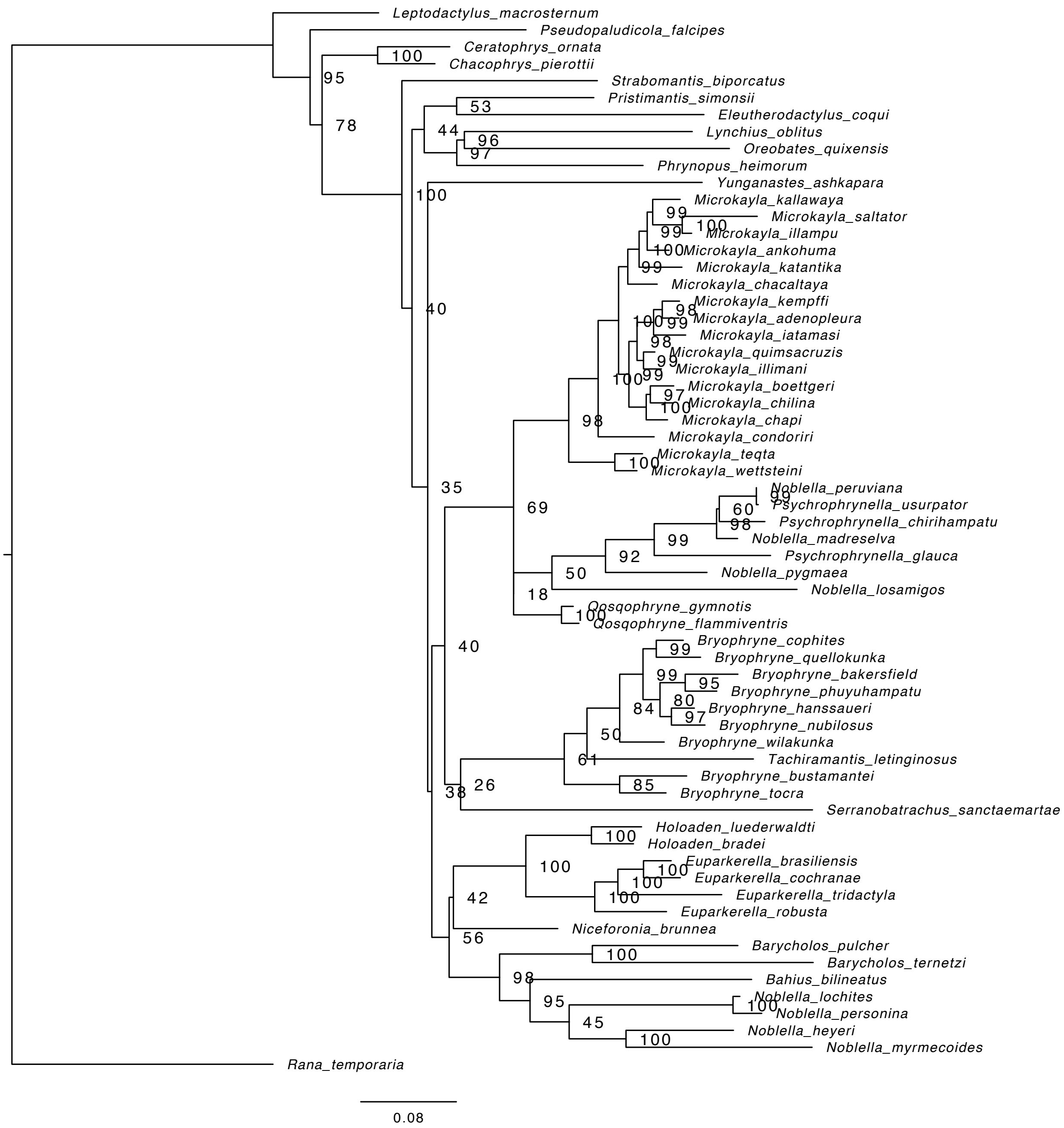

Figure S1: Phylogenetic relationships inferred by IQ-TREE concatenated analysis based on the DM dataset. UFBoot values are shown next to the branches.

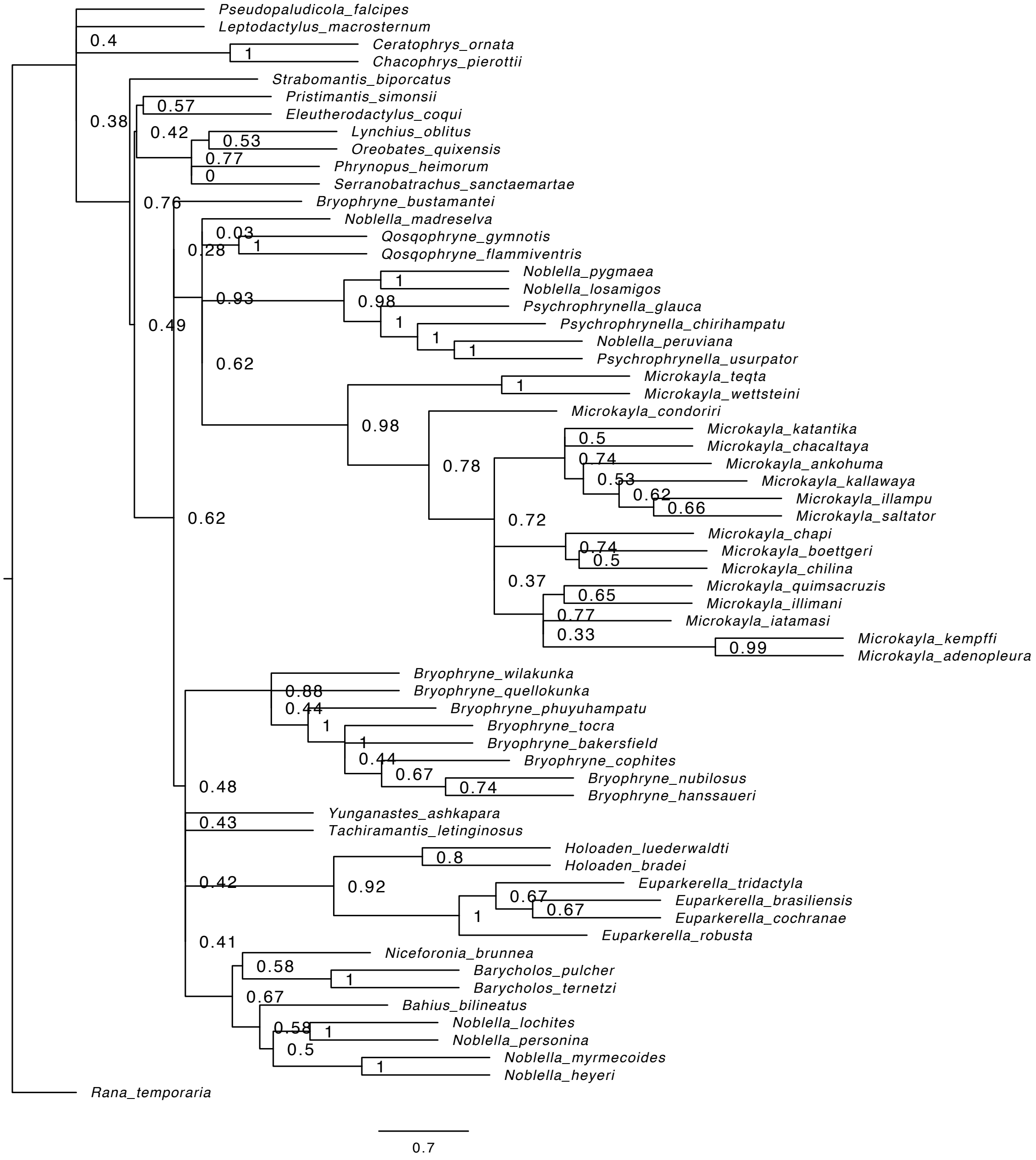

Figure S2: Phylogenetic relationships inferred by ASTRAL based on the DM dataset. Q1 support values are shown next to the branches.

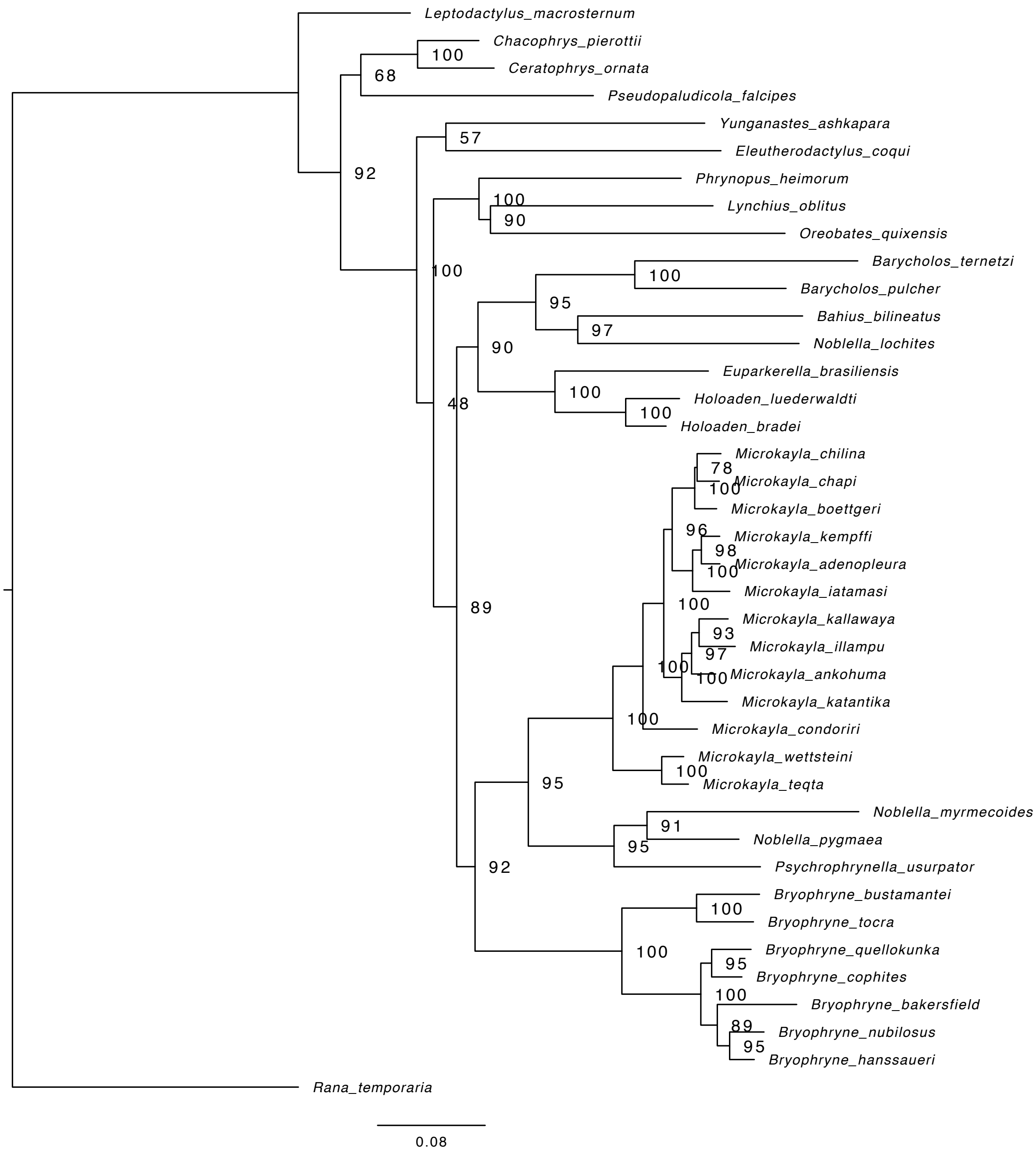

Figure S3: Phylogenetic relationships inferred by IQ-TREE concatenated analysis based on the D4 dataset. UFBoot values are shown next to the branches.

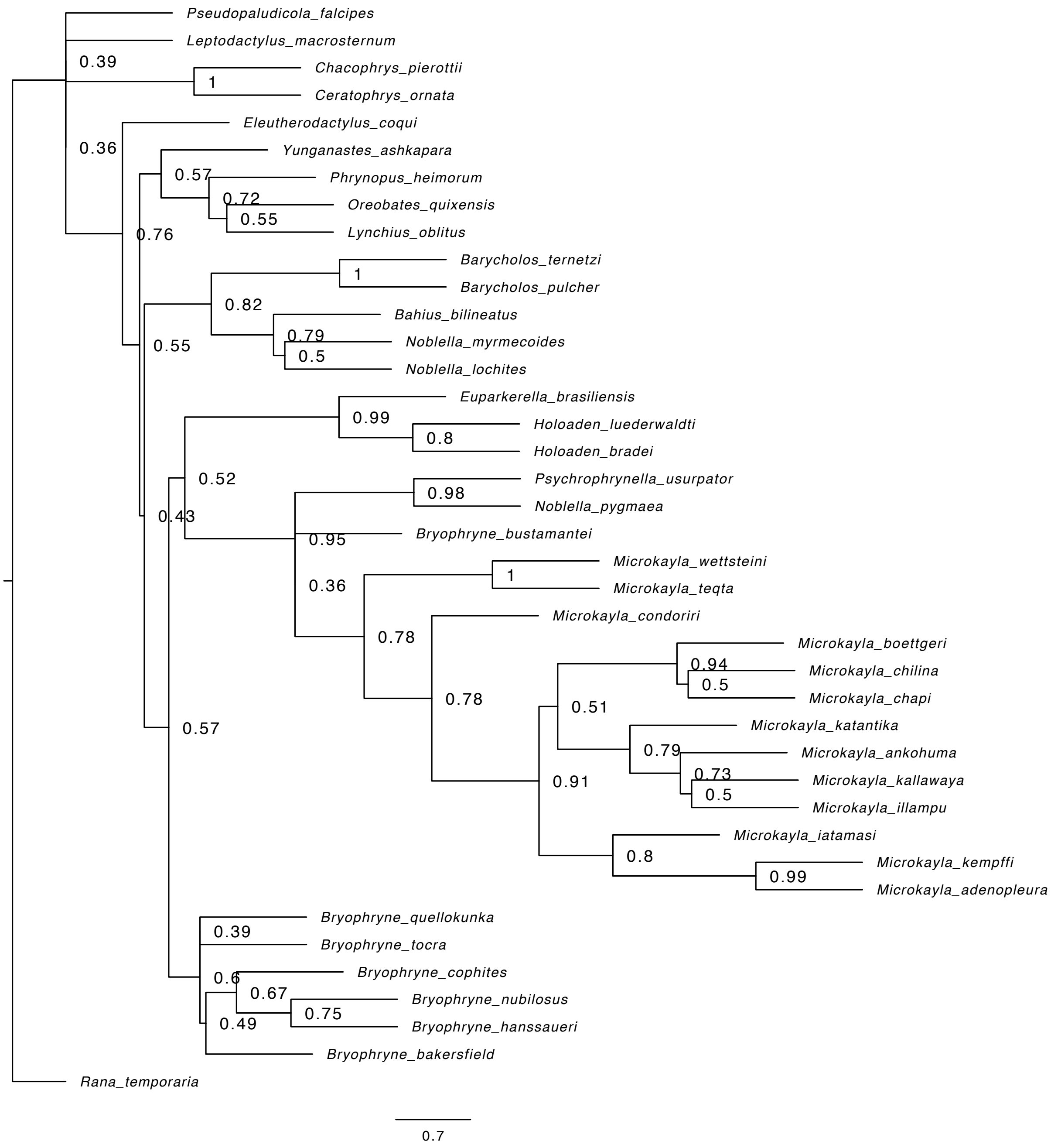

Figure S4: Phylogenetic relationships inferred by ASTRAL based on the D4 dataset. Q1 support values are shown next to the branches.

- Molecular dating

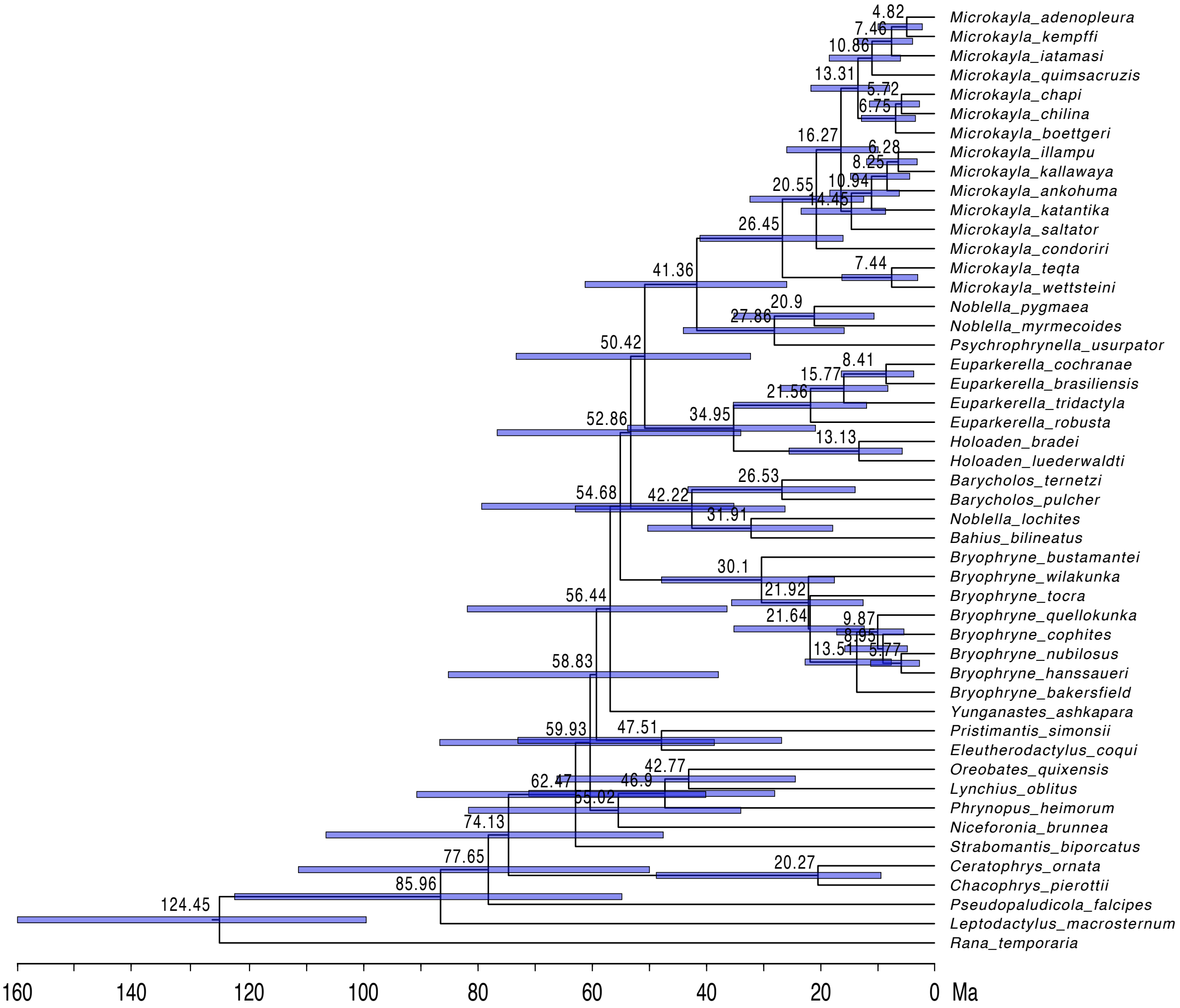

Figure S5: MCMCTree divergence time estimates based on the D3 dataset using an uncorrelated rates model and calibration scenario 1. HPD intervals are shown next to the nodes.

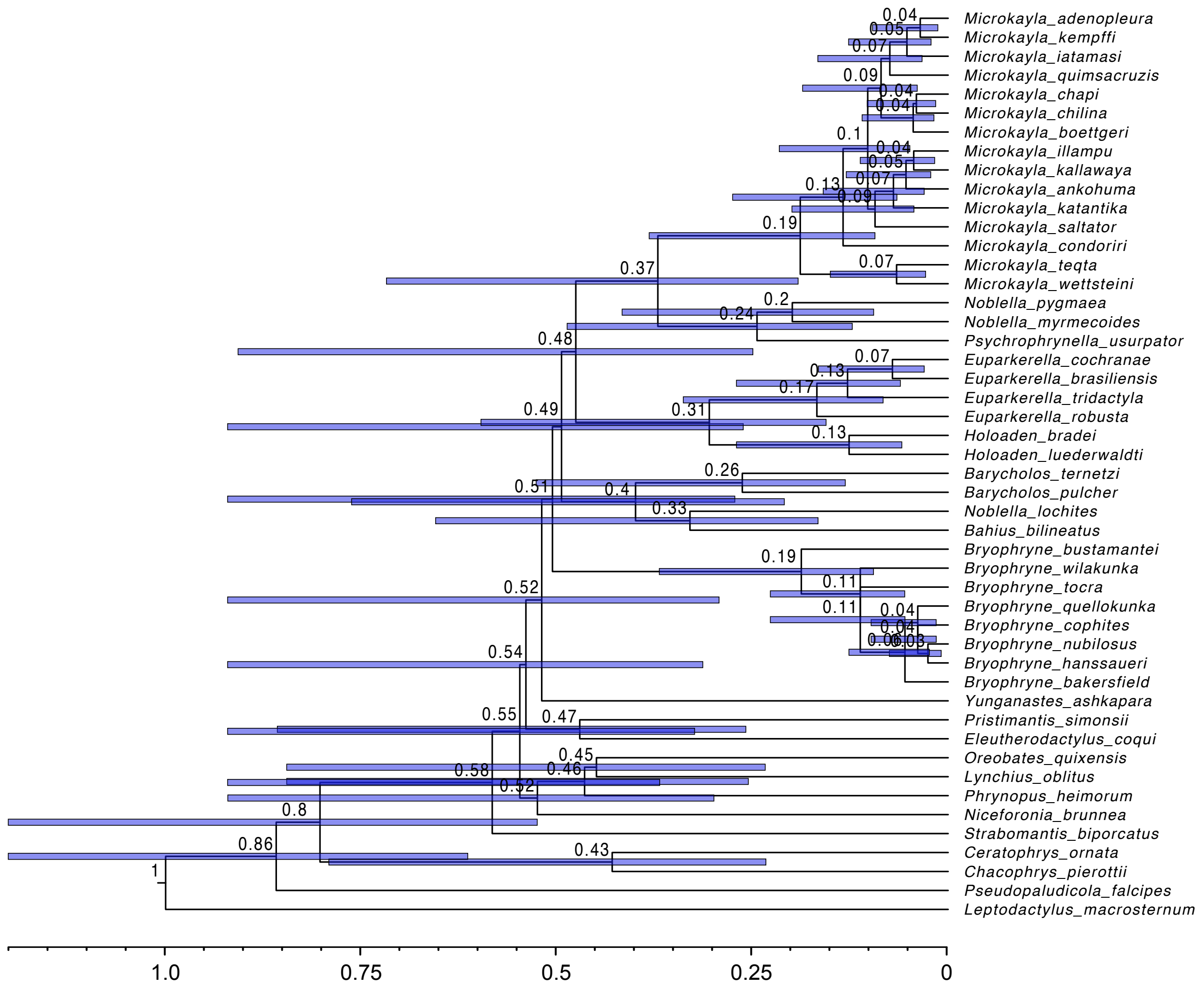

Figure S6: RelTime relative divergence time estimates based on the D3 dataset. No calibrations were used.

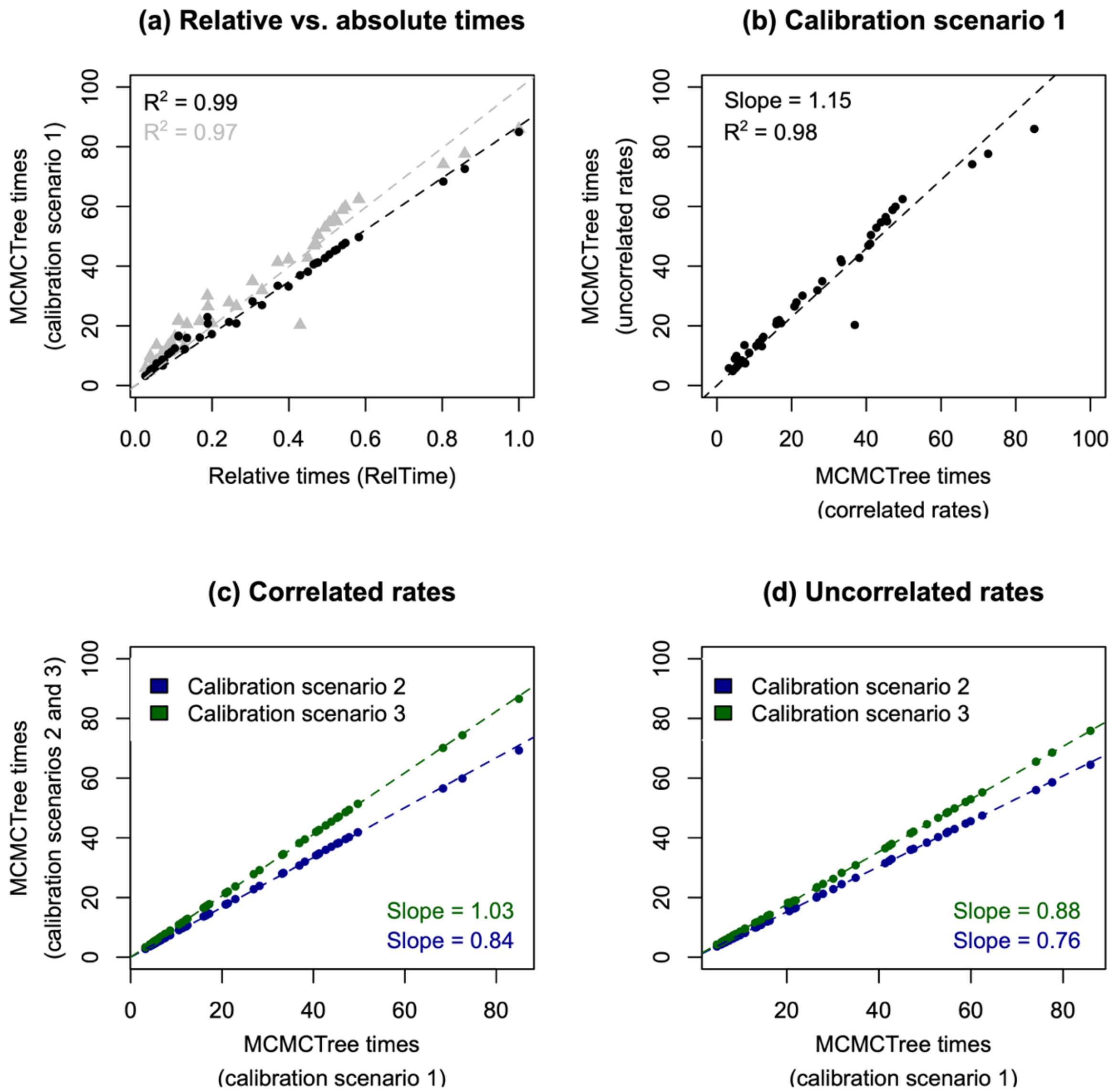

Figure S7: **(a)** Comparison of RelTime relative time estimates (without using calibrations, x-axis) and MCMCTree absolute time estimates (using calibrations, y-axis) (this is identical to Figure 2). MCMCTree estimates using a correlated rate evolution model are represented by black dots, while estimates using an uncorrelated rates model are depicted by gray triangles. The dashed lines indicate the linear regressions through the origin, considering correlated rates (black) and uncorrelated rates (gray). **(b)** Comparison of MCMCTree divergence times using a correlated rate evolution model (x-axis) and MCMCTree divergence times using an uncorrelated rate evolution model (y-axis), both under Calibration Scenario 1. The dashed line indicates the linear regression through the origin. **(c)** Comparison of MCMCTree divergence times using Calibration Scenario 1 (x-axis) and Calibration Scenario 2 and 3 (x-axis) under the correlated rate evolution model. The dashed lines indicate the linear regressions through the origin. **(d)** Comparison of MCMCTree divergence times using Calibration Scenario 1 (x-axis) and Calibration Scenario 2 and 3 (x-axis) under the uncorrelated rate evolution model. The dashed lines indicate the linear regressions through the origin.
